# Identification and Validation of Genomic Subtypes and a Prognostic Model Based on Antigen-Presenting Cells and Tumor Microenvironment Infiltration Characteristics in Hepatocellular Carcinoma

**DOI:** 10.3389/fonc.2022.887008

**Published:** 2022-06-03

**Authors:** Ji Chen, Chunxue Li, Zhichao Lang, Jianjian Zheng, Suhui Yu, Zhenxu Zhou

**Affiliations:** ^1^Key Laboratory of Clinical Laboratory Diagnosis and Translational Research of Zhejiang Province, The First Affiliated Hospital of Wenzhou Medical University, Wenzhou, China; ^2^Department of Hepatobiliary Surgery, The First Affiliated Hospital of Wenzhou Medical University, Wenzhou, China; ^3^Department of Hernia and Abdominal Wall Surgery, The First Affiliated Hospital of Wenzhou Medical University, Wenzhou, China

**Keywords:** antigen-presenting cells, hepatocellular carcinoma, genomic subtypes, prognostic model, immune infiltration

## Abstract

Currently, the prognosis of hepatocellular carcinoma (HCC) is poor, and there is a lack of effective targeted therapy. As key mediators of the immune response, the prognostic value of antigen-presenting cells (APCs) in HCC still remains unclear. In this study, we aimed to identify APC-related genomic subtypes and develop a novel prognostic model in HCC. Our results indicated that overall survival (OS) and the level of immune infiltration significantly differed between different APC clusters. By analyzing the gene expression profile between APC clusters, APC-related genomic subtypes were identified. There was a significant difference in OS and tumor microenvironment infiltration in HCC patients with different genomic subtypes. With the aid of genomic subtypes, significantly differentially expressed genes were screened to generate a novel prognostic model. The risk score of the model had a significant positive correlation with APCs and was associated with immune checkpoint expressions. Through the clinical cohort collected from the First Affiliated Hospital of Wenzhou Medical University, the prognostic value of the risk score was further validated. Moreover, after the risk score and clinical characteristics were combined, a nomogram was constructed to evaluate the prognosis for HCC patients. In conclusion, we mainly identified the APC-related genomic subtypes and generated a novel prognostic model to improve the prognostic prediction and targeted therapy for HCC patients.

## Introduction

Hepatocellular carcinoma (HCC), the fourth most frequently diagnosed cancer worldwide, accounts for about 90% of primary liver cancers (1). HCC contributes to approximately 800,000 deaths each year with the most common risk factors such as hepatotropic viruses (HBV and HCV), alcohol consumption, and aflatoxin B1 exposure (2). Some significant improvements in the therapy of HCC have been made over the past 2 or 3 decades, such as local ablative therapies, resection, or transplantation (3). Recently, immunotherapy for HCC has also been developed, which is expected to become a new approach for targeted therapy of HCC (4, 5). It has been reported that the biological processes of HCC are closely associated with immune cell subpopulations, such as T cells and macrophages (6). In addition, the tumor microenvironment (TME) has also been found to be closely related to the unfavorable prognosis and aggressive metastasis of HCC (7, 8). Currently, TME has been considered an important modulator of HCC development and progression and a source of identifying targets for potential therapeutic agents by offering, inhibiting, or stimulating growth signals (9). Taken together, exploring immune infiltration-related pathways contributes to improving the assessment of HCC prognosis and may provide new therapeutic avenues for HCC.

Antigen-presenting cells (APCs), as the critical initial process in the immune response, play a key role in presenting exogenous antigens on major histocompatibility complex class I (MHC I) (10). APCs are essential for the induction of protective cytotoxic T cell (CTL) responses against tumors and various viruses (11, 12). Among APCs, dendritic cells (DCs) are the most powerful APCs, which play a crucial role in stimulating immature T cells and initiating the primary immune response (13, 14). It is also known that macrophages and B cells play a key role in antigen presentation and immune response, contributing to the immune situation for cancers (15, 16). Currently, APCs have been found to be used as prognostic biomarkers in breast cancer and colon cancer (17, 18). Increasing evidence has been shown that APCs could serve as stratification factors to develop new therapeutic strategies for colorectal cancer (19). Recently, APCs have been found to be involved in the regulation of the immune environment of HCC (20). However, the prognostic value of APCs in HCC still remains largely unknown.

## Materials and Methods

### Data Preparation

In this study, a total of 365 HCC patients, including complete RNA transcriptome data (FPKM normalized) and clinical characteristics (including age, gender, tumor grade, T stage, N stage, and M stage), were obtained from The Cancer Genome Atlas (TCGA) database (https://portal.gdc.cancer.gov) as TCGA cohort. HCC patients from a local HCC cohort (n = 115), including clinical characteristics and antigen-presenting related gene (ARG) expressions, were enrolled at the First Affiliated Hospital of Wenzhou Medical University (FAHWMU) (Wenzhou, China). The collection of this cohort was reviewed and approved by the human research ethics committee of the FAHWMU. All patients/participants provided their written informed consent to participate in this study. The 17 ARGs (PNRC1, CTSS, CTSF, HLA-DRA, HLA-DRB1, ATF3, RFX3, USP11, USP34, PSMA3, PSMA4, IRF1, CD207, CLEC4A, CLEC5A, CD1A, and CD1C) used in this study were gained from MSigDB database (https://www.gsea-msigdb.org/gsea/msigdb).

Among the clinical characteristics in the FAHWMU cohort, the laboratory variables were taken from the results of a test closest to the date of surgery, including hepatitis B, alpha-fetoprotein (AFP), carcinoembryonic antigen (CEA), and carbohydrate antigen 19-9 (CA199). The histopathological variables (including tumor size, lymph node invasion, vascular invasion, and perineural invasion) were also included. Based on the 8th edition of the American Joint Committee on Cancer (AJCC) Staging Manual, the TNM stage for each HCC patient was obtained. In addition, age and gender were included as the demographic characteristics.

The total RNA from the liver tissues of the FAHWMU cohort was extracted using TRIzol reagent. The mRNA was then reverse transcribed into cDNA using ribo SCRIPTTM reverse transcription kit. The expression levels of mRNA were calibrated with glyceraldehyde-3-phosphate dehydrogenase (GAPDH). SYBR Green master mix was added, and real-time PCR was carried out using a 7500 rapid quantitative PCR system (Applied Biosystems, Foster City, CA, USA). The CT value of each well was recorded, and the relative quantification of the amplified products was performed using the 2^−ΔCt^ method.

### Enrichment Analysis

To initially analyze the related functionality of ARGs, the Gene Ontology (GO) enrichment analysis was performed according to the Enrich database (http://amp.pharm.mssm.edu/Enrichr/) (21). In addition, the Kyoto Encyclopedia of Genes and Genomes (KEGG) enrichment analysis, based on the DAVID database (https://david.ncifcrf.gov/), was utilized to reveal the ARG-related potential signaling pathways. Through the Genomics of Drug Sensitivity in Cancer (GDSC; https://www.cancerrxgene.org) dataset, the response of HCC patients to potential chemotherapeutic drugs was predicted. The sensitivity of chemotherapeutic drugs was calculated by the half-maximal inhibitory concentration (IC50) method, and IC50 was estimated using the R package “pRRophetic” (22). In targeting correlation analyses with respect to immune checkpoints, the Wilcoxon test and Pearson’s correlation analysis were applied to calculate the relationship between different groups.

Raw data on tumor mutational burden (TMB) for HCC patients were enrolled from TCGA database (https://portal.gdc.cancer.gov/). With the aid of the “mattool” R package, upstream analysis of whole-genome sequencing and whole-exome sequencing data was performed (23). For systematic analysis of TMB indicators in individual HCC patients, somatic mutation analysis was used, with the “getsamplesummary” function and the “getgenesumprice” function retrieving sample information and gene information, respectively (24).

### Consensus Clustering

The consensus clustering method was used to identify the APC-related subtypes in this study (25). In the consensus clustering, the resampling method was applied to reduce the error. Meanwhile, the number of cluster k values was specified to calculate the rationality under different cluster numbers (26). The k value with the smallest cumulative distribution function (CDF) slope was considered the optimal value for separating clusters when the consensus index value ranged from 0.1 to 0.9 (27).

### Estimation of Relative Immune Cell Content

The relative ESTIMATE score, Immune score, and Stromal score for individual HCC patients were estimated using the Estimation of STromal and Immune cells in MAlignant Tumor tissues using the Expression data (ESTIMATE) algorithm (28). Meanwhile, the proportion of 22 tumor-infiltrating immune cells (TICs) was calculated through the CIBERSOFT algorithm, which is a gene-based deconvolution algorithm that infers 22 human TIC types and quantifies the relative fraction of each cell type (29). The details for genes, which represent 22 TICs, are presented in **Table S1**. To assess the activity of the programmed death-ligand 1 (PD-L1) antibody in patients, the IMvigor210 trial was also applied (30).

### Clinical Implication Analysis

Kaplan–Meier (K-M) method was applied to construct K-M survival curves (31). Meanwhile, the log-rank test was used to validate the accuracy of the K-M survival curve (32). The receiver operating characteristic (ROC) curve, based on the R package “timeROC,” was generated to evaluate the correlation between indices and overall survival (OS) (33). The univariate Cox regression analysis was employed to further evaluate the clinical prognostic value. The predictors including clinical characteristics and risk scores were integrated by the nomogram. Through the Cox regression method, the calibration curve at the 1st, 2nd, and 3rd years and the PH hypothesis analysis were constructed to verify the accuracy of the nomogram **Table 1** (34).

**Table 1:** Clinical characteristics for HCC patients in TCGA cohort and the FAHWMU cohort.

	TCGA cohort (n = 365)	FAHWMU cohort (n = 115)
Age, years	61.19 ± 14.38	49.59 ± 11.38
Gender		
Male	246 (67.4%)	67 (58.3%)
Female	119 (32.6%)	48 (41.7%)
TNM stage		
I	170 (46.6%)	44 (38.3%)
II	84 (23.0%)	25 (21.7%)
III	83 (22.7%)	37 (32.2%)
IV	4 (1.1%)	9 (7.8%)
Unknown	24 (6.6%)	NA
Tumor size, cm		
≤5	NA	69 (60.0%)
>5	NA	46 (40.0%)
Hepatitis B		
Negative	NA	67 (52.3%)
Positive	NA	48 (41.7%)
Lymph node invasion		
No	NA	83 (72.2%)
Yes	NA	32 (27.8%)
Vascular invasion		
No	NA	84 (73.0%)
Yes	NA	31 (27.0%)
Perineural invasion		
No	NA	90 (78.3%)
Yes	NA	25 (21.7%)
Albumin, g/L	NA	39.00 ± 3.40
AFP, ng/ml	NA	2.52 ± 0.27
CEA, μg/L	NA	2.38 ± 0.57
CA199, U/ml	NA	46.80 ± 12.90

HCC, hepatocellular carcinoma; TCGA, The Cancer Genome Atlas; FAHWMU, First Affiliated Hospital of Wenzhou Medical University; AFP, alpha-fetoprotein; CEA, carcinoembryonic antigen; CA199, carbohydrate antigen 19-9; NA, Not Applicable.

## Results

### Antigen-Presenting Related Genes Were Closely Associated With the Progression of Hepatocellular Carcinoma

**Figure 1:**
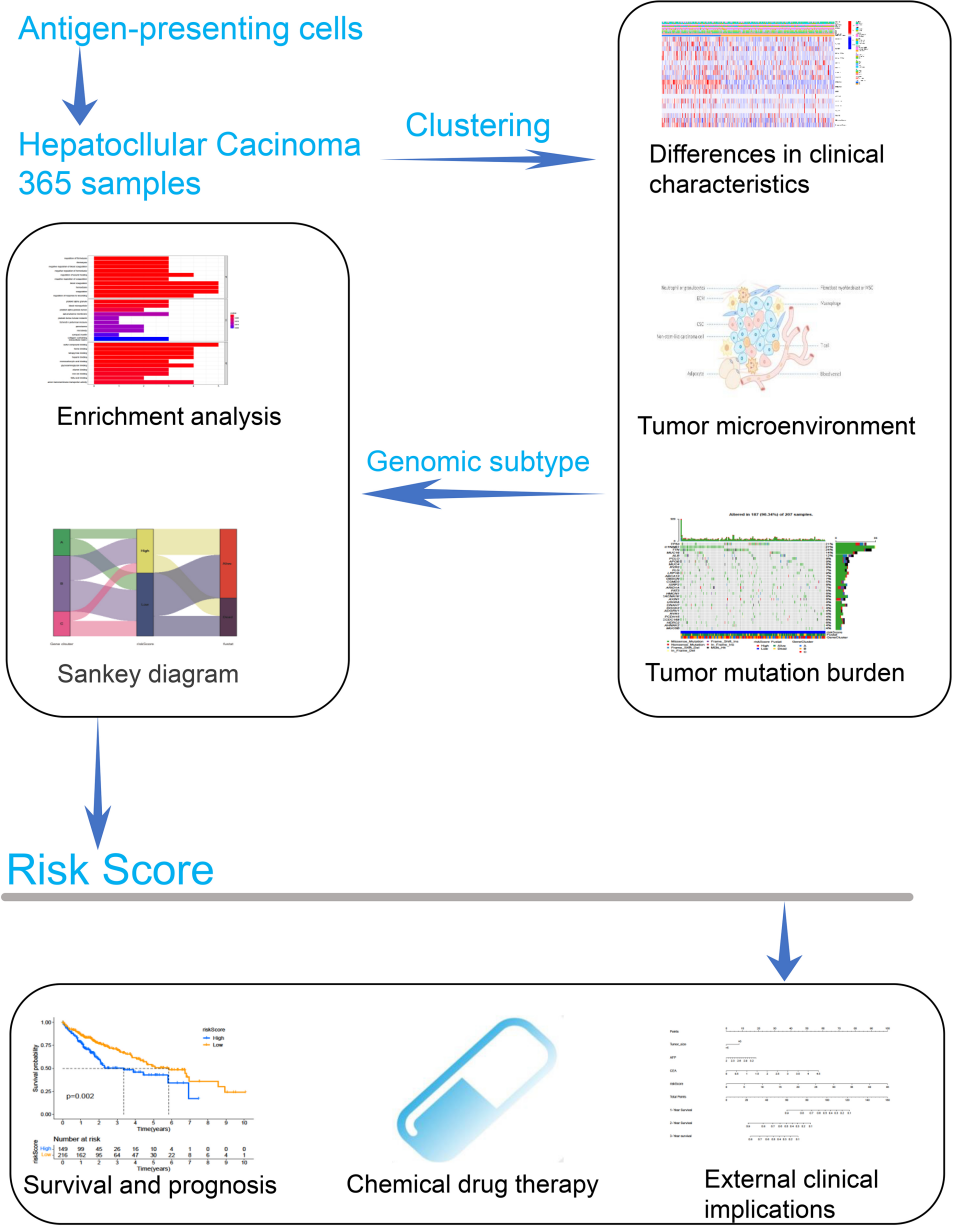
The overall workflow of this study.

**Figure 2:**
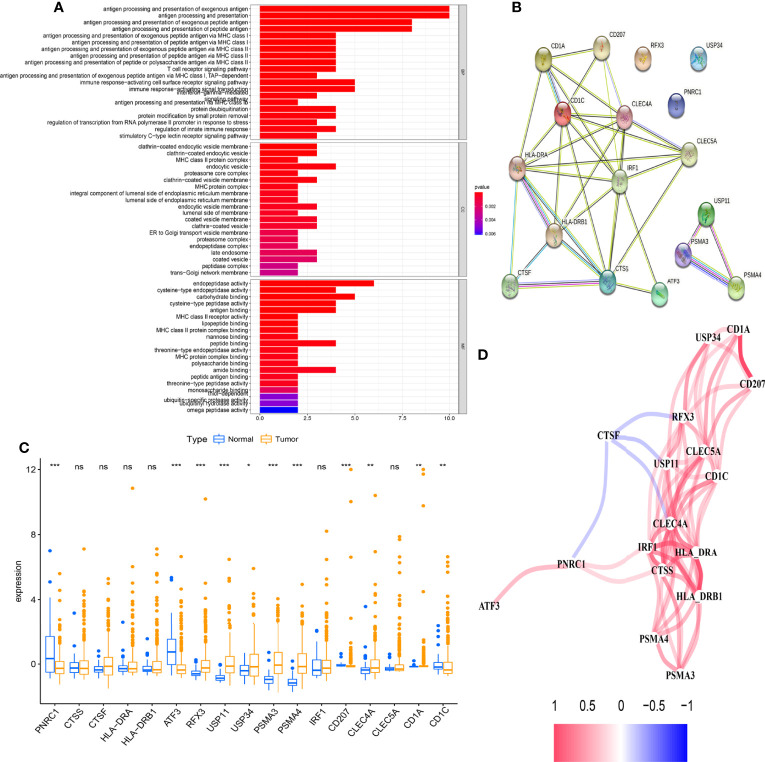
Differential expression of antigen-presenting cell-related genes (ARGs) is closely associated with the biological progression of hepatocellular carcinoma (HCC). **(A)** Gene Ontology (GO) enrichment analysis based on the differences in 17 ARG expression levels between adjacent non-tumorous samples and HCC samples (p < 0.05). **(B)** The protein-to-protein interaction (PPI) network of 17 ARGs (coef > 0.4). **(C)** The boxplot displays the expression differences of 17 ARGs between adjacent non-tumorous samples and HCC samples (*p < 0.05, **p < 0.01, ***p < 0.001). **(D)** The correlation network of ARGs. The correlation coefficients are represented by different colors. ns, no significance.

The overview of the study design is shown in **Figure 1**. As presented in **Figure 2A**, the GO function enrichment analysis revealed the ARG-enriched significant cellular functions. Except for the functions related to antigen presentation, there were also a large number of cellular functions closely related to various pathways of the immune response as well as stress response, protein ubiquitination, and interferon signature pathways. The protein-to-protein interaction (PPI) relationships of ARGs were displayed in the PPI network (**Figure 2B**). It was found that CD1C, IRF1, and CLEC4A had the most protein interactions with other ARGs. As indicated in **Figure 2C**, the ARG expressions were examined between adjacent non-tumorous samples and HCC samples. Our results showed that PNRC1, ATF3, CD207, and CD1C were obviously reduced in the HCC samples compared to non-tumorous samples, whereas RFX3, USP11, USP34, PSMA3, PSMA4, CLEC4A, and CD1A were increased in HCC samples. The correlation network of ARGs was presented in **Figure 2D**.

### The Clinical Value and Immune Characteristics for Antigen-Presenting Cell-Related Subtypes

**Figure 3:**
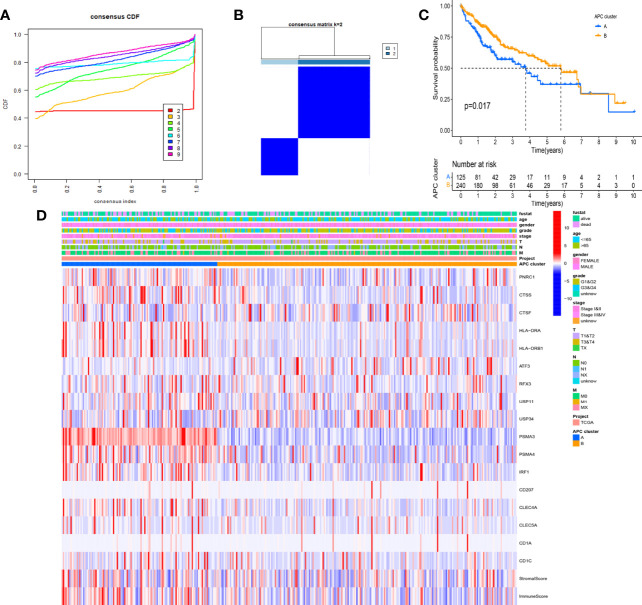
Identification of main antigen-presenting cell (APC)-related subtypes in hepatocellular carcinoma (HCC). **(A)** Identification of the optimal k value in the consensus clustering (k = 2). **(B)** Expression distribution of different subtypes when consumption index k = 2. **(C)** The Kaplan–Meier (K-M) survival curve of 2 main APC clusters (p = 0.017). **(D)** The complex heatmap displays the difference in clinical characteristics and ARG expression levels between different APC clusters.

**Figure 4:**
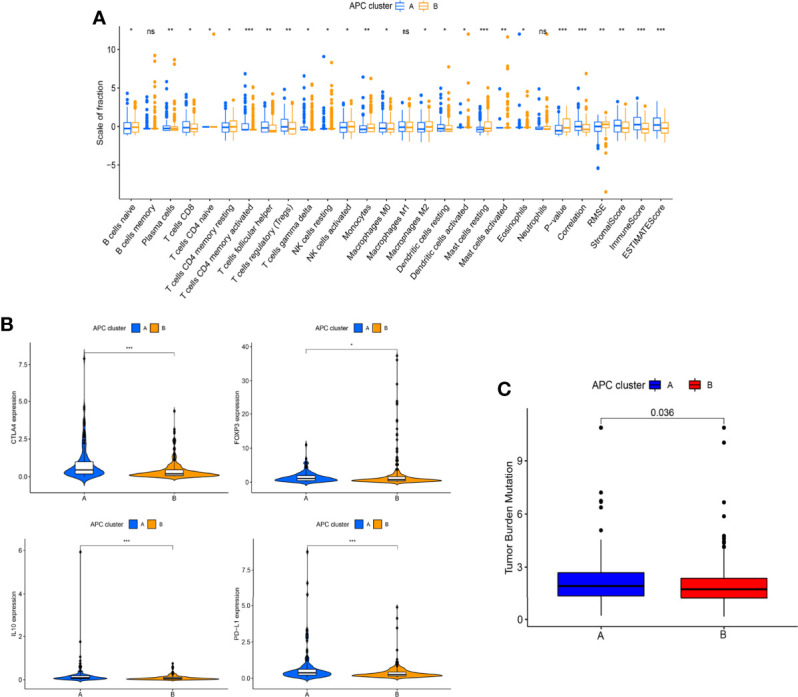
Differences in immune infiltration levels between different antigen-presenting cell (APC) clusters. **(A)** The boxplot shows the difference between 22 tumor-infiltrating immune cells (TICs) and ESTIMATE scores between different APC clusters. **(B)** Difference in the expression of 4 immune checkpoint between different APC clusters (*p < 0.05, **p < 0.01, ***p < 0.001). **(C)** Difference in tumor mutational burden (TMB) between different APC clusters (p = 0.036). ns, no significance.

Based on the CDF method with an optimal k value of 2, HCC patients in TCGA cohort were classified into 2 APC-related subtypes *via* consensus clustering analysis (**Figures 3A, B**, **Figure S1**). As shown in **Figure 3C**, the K-M survival curves showed that patients in the APC cluster A had a significantly lower OS compared to APC cluster B (**Figure 3C**, p = 0.017). The complex heatmap displayed the distribution of clinical characteristics and ARG expressions between APC clusters (**Figure 3D**). Most ARGs showed higher expression levels in APC cluster A in comparison with APC cluster B. As shown in **Figure 4A**, the relative scale of a fraction of plasma cells, T-cell CD4 memory activated, T-cell follicular helper, and T-cell regulatory (Tregs) was significantly downregulated in APC cluster B compared to APC cluster A (p < 0.01). By contrast, monocytes, mast cell resting, and mast cells activated were remarkably upregulated in APC cluster B (p < 0.01). Moreover, the TME scores (Immune score, Stromal score, and ESTIMATE score) were obviously lower in APC cluster B (p < 0.01). For TME scores, lower Immune score and Stromal score indicated a lower relative content of stromal cells and immunocytes in TME, while a lower ESTIMATE score indicated the lower aggregation of Stromal and Immune scores in TME. The associations between APC clusters and immune checkpoints were next analyzed. All immune checkpoints including CTLA4, FOXP3, IL10, and PD-L1 were obviously downregulated in APC cluster B (**Figure 4B**, p < 0.05), implying that the immune system of patients with APC cluster A may be suppressed. In addition, a lower TMB value was shown in APC cluster B (**Figure 4C**, p = 0.036), indicating that patients in APC cluster A may benefit from immunotherapy.

### The Clinical Value and Immune Characteristics of Genomic Subtypes

**Figure 5:**
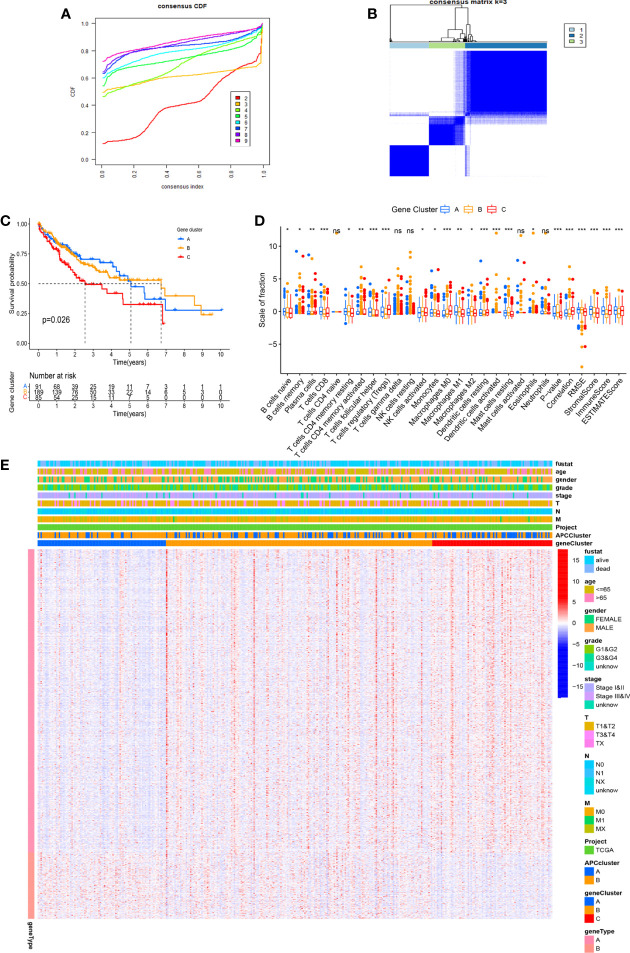
Identification of main genomic subtypes according to the antigen-presenting cell (APC) clusters. **(A)** Identification of the optimal k value in the consensus clustering (k = 3). **(B)** Expression distribution of different subtypes when consumption index k = 3. **(C)** The Kaplan–Meier (K-M) survival curve of 3 genomic clusters (p = 0.026). **(D)** The boxplot displays the difference between 22 tumor-infiltrating immune cells (TICs) among different genomic subtypes. **(E)** The complex heatmap shows the difference in clinical characteristics and differentially expressed gene (DEG) expression levels among different APC clusters. *p < 0.05, **p < 0.01, ***p < 0.001. ns, no significance.

**Figure 6:**
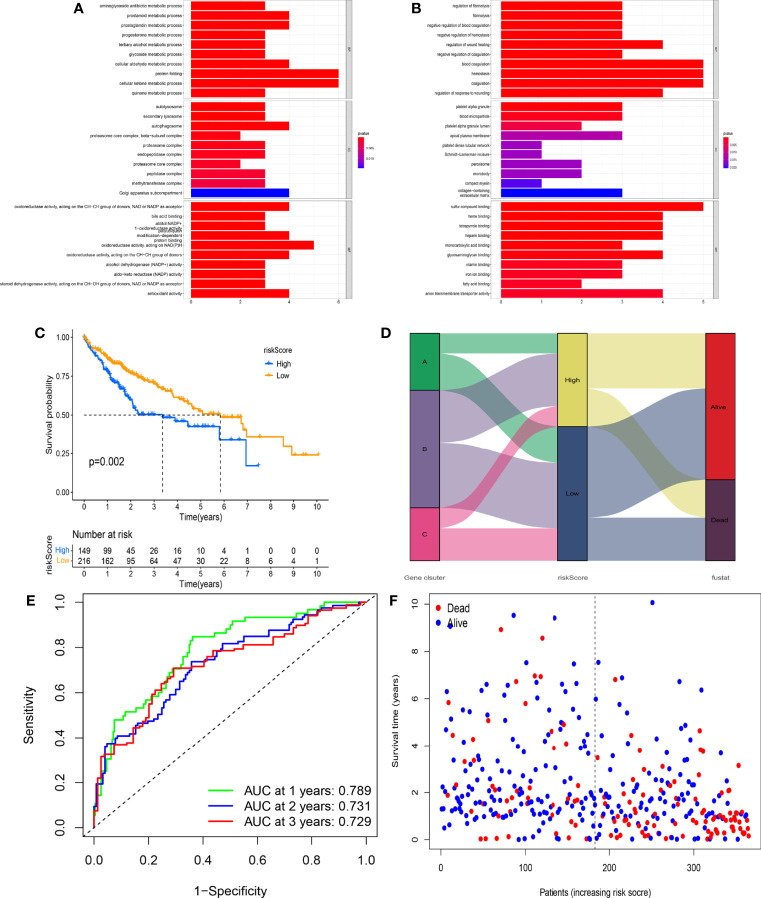
Prognostic value of the novel risk score. **(A)** Gene Ontology (GO) enrichment analysis based on the gene expression differences among different genomic subtypes (p < 0.05). **(B)** GO enrichment analysis based on the gene expression differences among different genomic subtypes (p < 0.05). **(C)** The Kaplan–Meier (K-M) survival curve displays the difference in overall survival (OS) between different risk scoring groups. **(D)** The correlation between genomic subtypes, risk score, and survival status. **(E)** The time-independent receiver operating characteristic (ROC) curve of the risk score regarding OS. **(F)** The association of the risk score, survival time, and survival status.

With the use of the Wilcoxon test, a total of 980 significantly differentially expressed genes (DEGs) were screened in APC clusters as the potential genes targeting APCs. Subsequently, the consensus clustering was performed again based on the genome matrix for 980 DEGs. Based on the CDF method, the optimal k value was selected to divide HCC patients into 3 main APC-related genomic subtypes (**Figures 5A, B**, **Figure S1**). The K-M survival curve was used to analyze the OS among genomic clusters. Our data indicated that genomic cluster C had lower OS compared to genomic clusters A and B (**Figure 5C**, p = 0.026). We additionally analyzed the scale of fraction for 22 main TICs and ESTIMATE scores among genomic subtypes (**Figure 5D**). The relations between clinical characteristics and DEGs expressions in different genomic subtypes are shown in the heatmap (**Figure 5E**). The expression of most DEGs tended to increase as APC-related clustering from A to C. The GO enrichment analysis showed the functions of immune responses, multiple metabolic responses, and various basic physiological activities differed significantly among genomic subtypes (**Figures 6A, B**, p < 0.05).

### Generation and Validation of the Prognostic Model

Based on the Lasso-Cox regression analysis of DEGs, a total of 12 hub prognostic-related genes were screened to generate the prognostic model. The risk score under the prognostic model was next calculated according to the gene expressions and optimal coefficients.

Risk score = EIF2S1 * 0.0087

+YBX1 * 0.0011

+OLA1 * 0.0022

+TRMT10C * 0.0032

+KIF20A * 0.0075

+SF3B4 * 0.0047

+ETF1 * 0.0024

+CDCA8 * 0.013

+YARS1 * 0.029

+DLAT * 0.0034

+TIMM23 * 0.0056

+GDI2 * 0.0020

All HCC patients in TCGA cohort were divided into the high-risk group (n = 149) and low-risk group (n = 216) according to the optimal risk score. The expression levels of 12 genes between risk groups were presented in **Figure S3A** (all p < 0.001). Interestingly, the high-risk group had lower OS as compared to the low-risk group (**Figure 6C**, p = 0.002). The distribution of genomic subtypes, risk scoring groups, and OS status is shown in **Figure 6D**. The time-independent ROC curve further indicated the predicting accuracy of the prognostic model in the 1st, 2nd, and 3rd years (**Figure 6E**, all areas under the curve (AUCs) > 0.700). The AUC value was significantly higher as compared to 4 published signatures (**Figure S2**) (35–38). The distribution of risk score, OS status, and OS time is shown in **Figure 6F**. Notably, with the increasing risk score, the OS time of most HCC patients tended to be downregulated, which further verified the prognostic value of the model.

**Figure 7:**
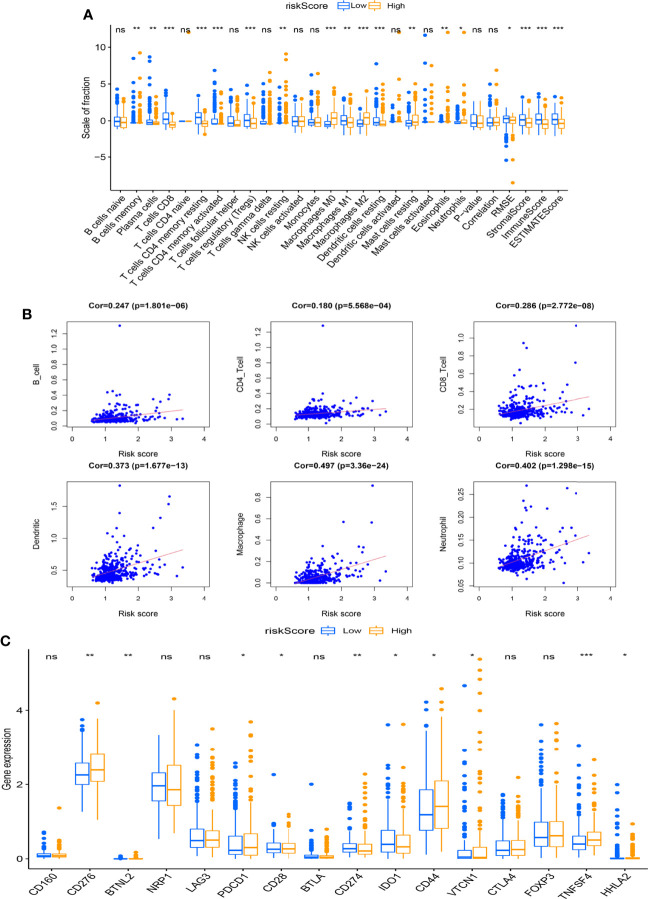
Association between the risk score and immune infiltration. **(A)** The boxplot displays the differences in tumor-infiltrating immune cell (TIC) expressions and ESTIMATE score between different risk scoring groups (*p < 0.05, **p < 0.01, ***p < 0.001). **(B)** The scatter chart shows the correlation between the risk score and TICs. **(C)** The boxplot indicates the differences of immune checkpoint expressions between different risk scoring groups (*p < 0.05, **p < 0.01, ***p < 0.001). ns, no significance.

It was found that the relative scale of fraction for plasma cells, T-cell CD8, T-cell CD4 memory resting, T-cell CD4 memory activated, Tregs, macrophages M1, and DCs resting was obviously lower in the high-risk group than that in the low-risk group (**Figure 7A**, p < 0.01). By contrast, macrophages M0 and M2 were remarkably higher in the high-risk group than in the low-risk group (p < 0.01). In addition, all the ESTIMATE scores were significantly downregulated in the high-risk group (p < 0.001). We also analyzed the correlation between risk score and the relative levels of immune infiltration (**Figure 7B**). There were positive correlations between immune cells (B cells, CD4 cells, CD8 cells, DCs, macrophages, and neutrophils) and risk score. Further studies were performed to explore the relations between immune checkpoint expressions and risk scores (**Figure 7C**).

**Figure 8:**
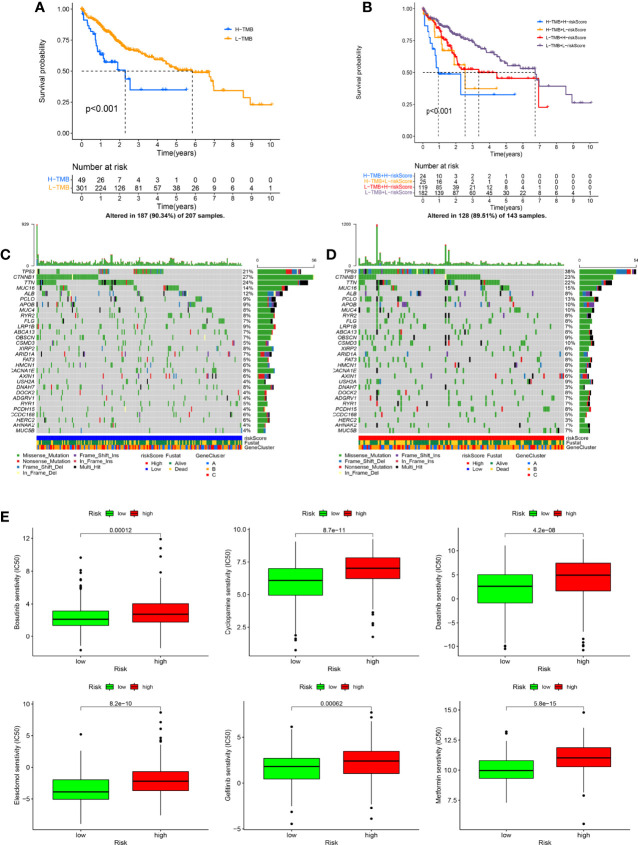
The exchange of risk score is closely related to tumor mutational burden (TMB) and drug sensitivity. **(A)** The Kaplan–Meier (K-M) survival curve shows the difference in overall survival (OS) in the different TMB groups. **(B)** The K-M survival curve displays the combined impact of TMB and risk score on the OS. **(C, D)** The oncoplots indicate the differences in TMB status (including mutation type, number of mutations, and mutated genes) between risk scoring groups. **(E)** The drug sensitivity analysis shows the correlation between risk score and chemotherapeutic drugs (including bosutinib, cyclopamine, dasatinib, gefitinib, metformin, and elesclomol).

Based on the optimal TME value calculated by the mattools methods, all HCC patients were assigned to the high-TMB (n = 49) and the low-TMB groups (n = 301). The results of the K-M survival curve indicated that the OS rate for HCC patients with high TMB was significantly worse than that with low TMB (**Figure 8A**, p < 0.001). The prognostic value of risk score combined with TMB is shown in **Figure 8B**. We found that patients with low-TMB and low-risk scores had remarkably better prognostic status than others (p < 0.001). In addition, the oncoplots demonstrated the differences in mutated genes, mutation frequency, mutation types, and OS status between risk scoring groups (**Figures 8C, D**). **Figure 8E** indicates the results of the drug sensitivity test between risk score and six common chemotherapeutic drugs (including bosutinib, cyclopamine, dasatinib, gefitinib, metformin, and elesclomol) targeting HCC. Taken together, the risk score showed significant sensitivities to all chemotherapeutic drugs (p < 0.001). In the IMvigor210 cohort, the risk score of patients with stable disease or progressive disease (SD/PD) was remarkably lower than that of patients with complete response or partial response (CR/PR) (**Figure S3B**, p = 0.004).

### The Clinical Prognostic Value of the Risk Score Was Verified in the External Cohort

**Figure 9:**
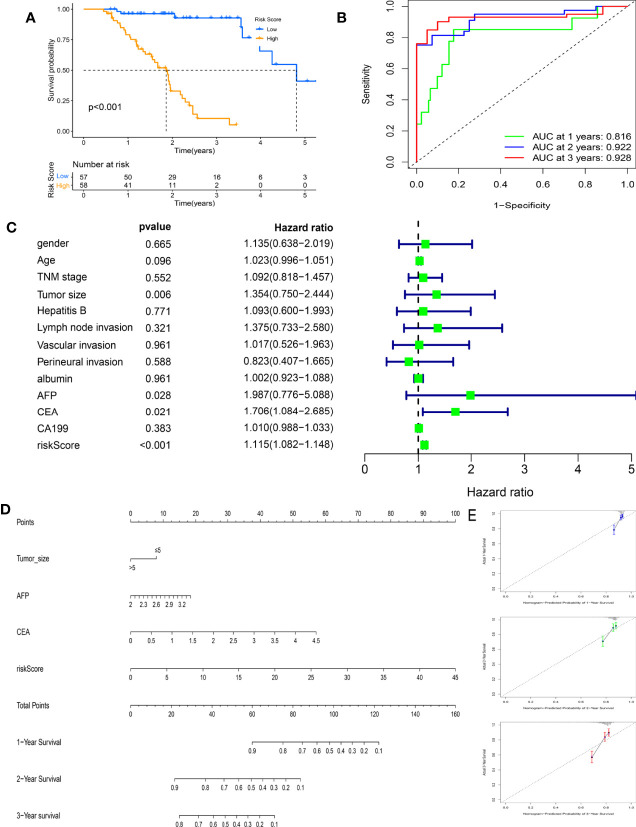
Clinical prognostic value of the risk score in the First Affiliated Hospital of Wenzhou Medical University (FAHWMU) cohort (n = 115). **(A)** The Kaplan–Meier (K-M) survival curve indicates the difference in overall survival (OS) in the different risk scoring groups. **(B)** The time-independent receiver operating characteristic (ROC) curve of the risk score. **(C)** The univariate Cox regression analysis of the risk score and clinical characteristics (including gender, age, TNM stage, tumor size, hepatitis B, lymph node invasion, vascular invasion, perineural invasion, albumin, alpha-fetoprotein (AFP), carcinoembryonic antigen (CEA), and carbohydrate antigen 19-9 (CA199)) regarding OS. **(D)** The nomogram combines the risk score and clinical characteristics significantly related to OS (p < 0.05). **(E)** The calibration curves are used to verify the nomogram in the 1st, 2nd, and 3rd years.

The clinical prognostic value of this prognostic model was further validated in the FAHWMU cohort (n = 115). According to the above calculation formula, all HCC patients in the FAHWMU cohort were separated into the high-risk (n = 58) and low-risk groups (n = 57). The K-M survival curve indicated that the risk score had a good performance for predicting the OS outcomes (**Figure 9A**, p < 0.001). The time-independent ROC curve further demonstrated the prognostic value of the model (**Figure 9B**). It was found that the AUC value reached 0.816 in the 1st year, 0.922 in the 2nd year, and 0.928 in the 3rd year. The univariate Cox regression analysis of the risk score and clinical characteristics (including gender, age, TNM stage, tumor size, hepatitis B, Lymph node invasion, vascular invasion, perineural invasion, albumin, AFP, CEA, and CA199) was performed. Clearly, it was found that risk score, tumor size, AFP, and CEA were remarkably correlated with OS of HCC patients (**Figure 9C**, p < 0.05). Subsequently, they were used to construct a novel nomogram to better predict the prognosis of HCC (**Figure 9D**). In addition, the calibration curves were applied to validate the accuracy of the nomogram in the 1st, 2nd, and 3rd years (**Figure 9E**). The PH hypothesis analysis of the nomogram model is shown in **Table S2**.

## Discussion

Recent studies have demonstrated that mining the subtypes of HCC patients through bioinformatics ways could contribute to identifying prognostic patterns for HCC (39, 40). Wu et al. previously defined a CPS1-deficient HCC subtype to provide new insights for therapeutics of HCC through targeting fatty acid oxidation (41). Currently, new tumor immunotherapies based on the APCs have also been explored, which are expected to become new approaches for targeted therapy of HCC (42). Kirkin et al. found that tumor-reactive effector cells can be generated *in vitro* by exposure to antigens induced by DNA demethylation (43). The tumor-reactive effector cells can be considered APCs to provide a novel invasive therapeutic strategy for treating cancer. Chiozzini et al. demonstrated that tumor cells with professional antigen-presenting functions resulted in the induction of effective tumor-specific CTL immune responses (44). Recently, it has been reported that programmed cell death ligand 1 (PD-L1) expressed on APCs plays a crucial role in checkpoint blockade therapy for HCC (45). Currently, there is still huge potential for the development of HCC immunotherapy targeting APCs. In this study, we identified APC-related genomic subtypes of HCC to determine their prognostic patterns and explore immunotherapy methods. In addition, a novel prognostic model was generated to improve the prognostic prediction of HCC.

Previous studies have found that by assessing immunosuppressive factors and immunomodulatory effects of APCs, immunosuppressive TME is associated with the effects of cancer immunotherapy (46). The activation of APCs can elicit the TME to generate a strong immune response (47). In this study, APC-related subtypes were closely related to TME scores and the relative contents of TICs, especially for T cells and macrophages. With the increase of the risk score in the prognostic model, the ESTIMATE scores for TME and relative contents of T cells tended to be downregulated, while the relative contents of macrophages tended to be upregulated. In fact, it has been found that APCs pulsed with gp96-peptide complexes derived from HCC cells are effective in activating specific T-cell responses (48). In addition, ARGs could be highly enriched in macrophages, leading to the increased infiltration of CD8 cytotoxic T lymphocytes, which significantly affects the immune environment of HCC (49). Consistent with the previous findings, our results also provide new insights into the identification of the immune environment of HCC.

Among 12 hub genes in our risk signature, GDI2 is mainly involved in the lipid metabolism and extracellular matrix (ECM) building pathways of HCC (50). DLAT may serve as a potential immunotherapeutic target for its affection to cancer cell proliferation and carbohydrate metabolism (51). Previous studies showed that targeting CDCA88 may be the next molecular strategy for primary HCC treatment and prevention of metastasis or recurrence (52). Activation of ETF1 could effectively increase cancer cell invasion and metastasis, which may provide new insights for developing therapeutic strategies (53). Negatively regulated by miRNA-133b, SF3B4 has also been found to effectively promote cell proliferation and metastasis in HCC. The miRNA-133b/SF3B4 axis may serve as a new immunotherapeutic target for HCC treatment (54). The other hub genes (e.g., YBX1, OLA1, TRMT10C, and KIF20A) also play a key role in the development and prognosis of the pan-cancer section (55–58). Meanwhile, the role of TIMM23, YARS1, and EIF2S1 in affecting HCC is still unclear. Herein, the combined clinical value of these genes was further explored.

Currently, immune checkpoint inhibitor (ICI) therapy targeting anti-PD-1 or its ligand anti-PD-L1 is a key step in combination regimens aimed at improving the prognosis of HCC patients (59). Recent studies have reported that combining anti-PD-L1 with anti-CTLA4 increased the function of tumor-infiltrating lymphocytes and restored HCC-derived T-cell responses to tumor antigens (60). In line with it, our results indicated that the expression of PD-1 (CD274) was significantly lower in the high-risk group, whereas the expressions of CD276 and TNFSF4 had the opposite effects. These results were consistent with the distribution of immune checkpoints, and TNFSF4 may serve as a potential target for the immunotherapy of HCC. Among the chemotherapeutic drugs used in this study, dasatinib could specifically reduce the viability of sr-HCC cells with upregulated activity for targeted therapy of HCC (61). Also, gefitinib is effective in promoting decreased cell proliferation and enhanced apoptosis in the human HCC cell line (62). In this study, the risk score showed remarkable sensitivity to these chemotherapeutic drugs, indicating its ability to serve as an indicator of the progression of HCC therapy treatment. It is known that nomograms are important for assisting treatment decisions and optimizing treatment approaches (63, 64). Meanwhile, it has been reported that the level of CA199 and AFP can be used as prognostic markers of HCC and accurately predict the changes of OS (65). We further combined CA199, AFP, the prognostic model, and other clinical characteristics into a nomogram, contributing to improving the prognostic prediction of HCC.

The advantages of this study could be summarized in the following aspects. It is the first time to determine the APC-related genomic subtypes and identify prognostic patterns and immune characteristics for HCC. Moreover, a novel prognostic model was constructed, contributing to the evaluation of the immune environment for HCC patients. In addition, the external clinical implication of the prognostic model was further validated in the FAHWMU cohort.

In conclusion, we identify the APC-related genomic subtypes, which contribute to determining prognostic patterns and characterization of TME infiltration of HCC. Based on the APC-related genomic subtypes, a novel prognostic model is generated to further improve the prognostic prediction and targeted therapy for HCC patients.

## Data Availability Statement

The datasets presented in this study can be found in online repositories. The names of the repository/repositories and accession number(s) can be found in the article/**Supplementary Material**.

## Ethics Statement

The collection of this cohort was reviewed and approved by the human research ethics committee of the First Affiliated Hospital of Wenzhou Medical University. All patients/participants provided their written informed consent to participate in this study.

## Author Contributions

ZZ and JC designed the study and analyzed the data. CL and ZL wrote the manuscript and revised the images. JZ and SY performed the literature search and collected data for the manuscript. All authors listed have made a substantial, direct, and intellectual contribution to the work and approved it for publication.

## Funding

The study was supported by Wenzhou Science and Technology Bureau (grant no. Y20210180).

## Conflict of Interest

The authors declare that the research was conducted in the absence of any commercial or financial relationships that could be construed as a potential conflict of interest.

## Publisher’s Note

All claims expressed in this article are solely those of the authors and do not necessarily represent those of their affiliated organizations, or those of the publisher, the editors and the reviewers. Any product that may be evaluated in this article, or claim that may be made by its manufacturer, is not guaranteed or endorsed by the publisher.

## Supplementary Material

Supplementary Figure 1 **(A)** CDF curves for selecting optimal antigen-presenting cells related subtypes. **(B)** CDF curves for selecting optimal APCs related genomic subtypes.

Supplementary Figure 2 ROC curves of 4 published signatures in the TCGA cohort.

Supplementary Figure 3 **(A)** The expression levels of 12 hub genes between risk groups. **(B)** The comparison of risk score for patients with stable disease or progressive disease (SD/PD) and patients with complete response or partial response (CR/PR) in the IMvigor210 cohort.

## Abbreviations

HCC, hepatocellular carcinoma; TCGA, The Cancer Genome Atlas; APCs, antigen-presenting cells; FDR, false-discovery rate; OS, overall survival; AUC, area under the curve; ROC, receiver operating characteristic; K-M, Kaplan–Meier; GO, Gene Ontology; CDF, cumulative distribution function; TME, tumor microenvironment; FAHWMU, First Affiliated Hospital of Wenzhou Medical University; AFP, alpha-fetoprotein; CEA, carcinoembryonic antigen; CA199, carbohydrate antigen 19-9; PPI, protein-to-protein interaction
