# Supplementary figures and images for: Identification and Validation of Genomic Subtypes and a Prognostic Model Based on Antigen-Presenting Cells and Tumor Microenvironment Infiltration Characteristics in Hepatocellular Carcinoma
Source: Front Oncol. 2022 Jun 3;12:887008. doi: 10.3389/fonc.2022.887008 (PMC9205444; doi:10.3389/fonc.2022.887008)

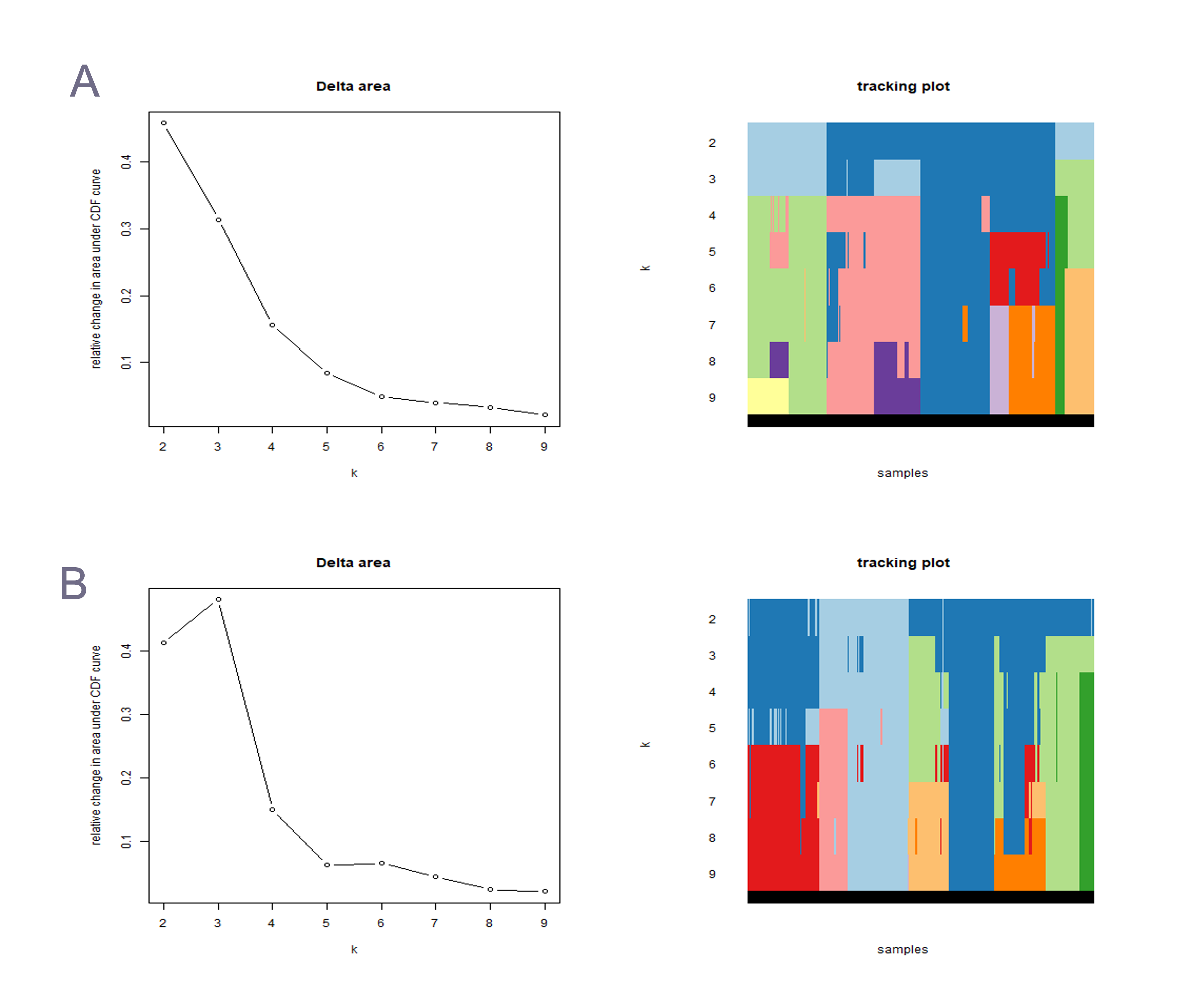

Supplement: Supplementary Figure 1 — (A) CDF curves for selecting optimal antigen-presenting cells related subtypes. (B) CDF curves for selecting optimal APCs related genomic subtypes. [file Image_1.tif]

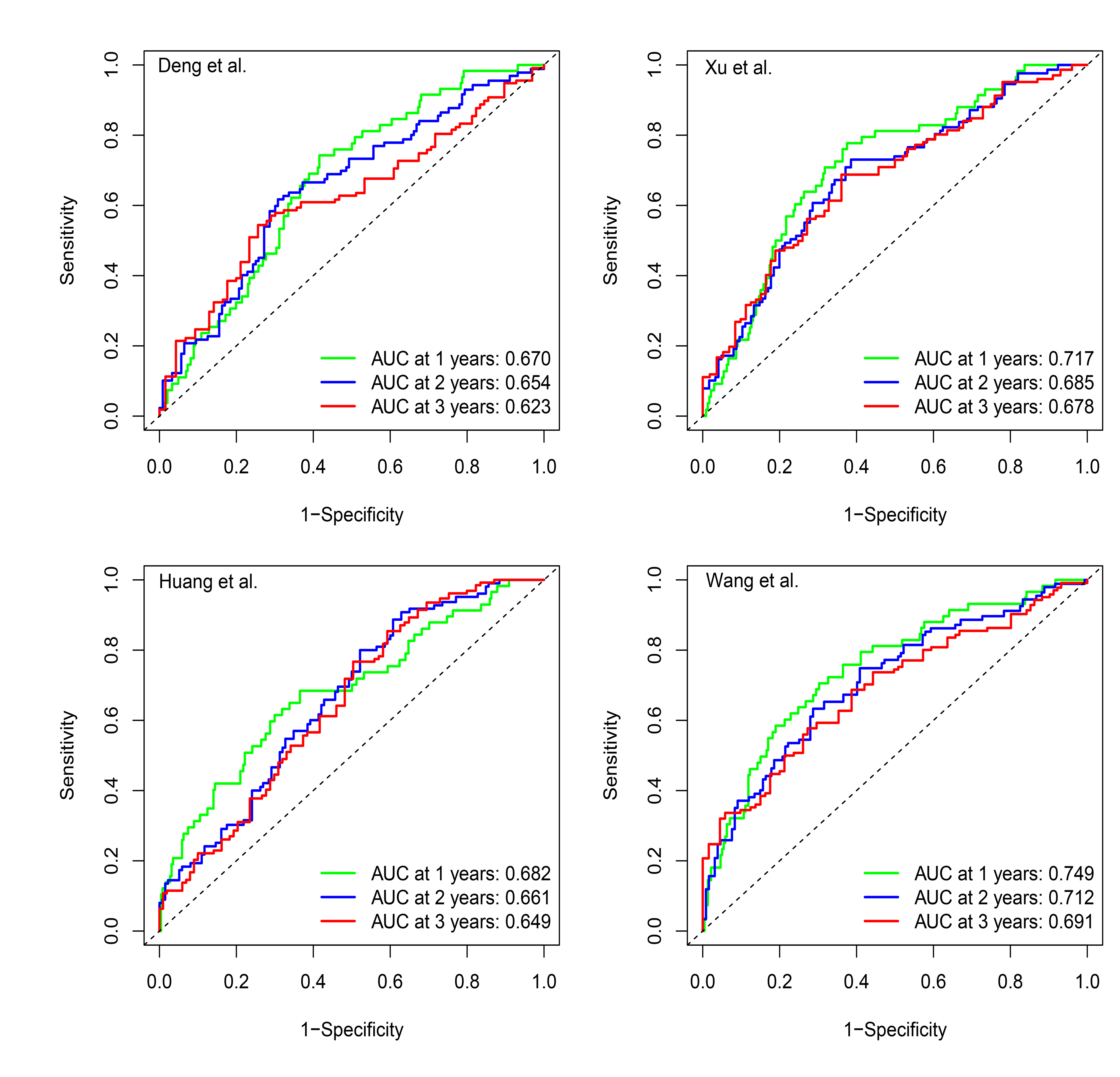

Supplement: Supplementary Figure 2 — ROC curves of 4 published signatures in the TCGA cohort. [file Image_2.tif]

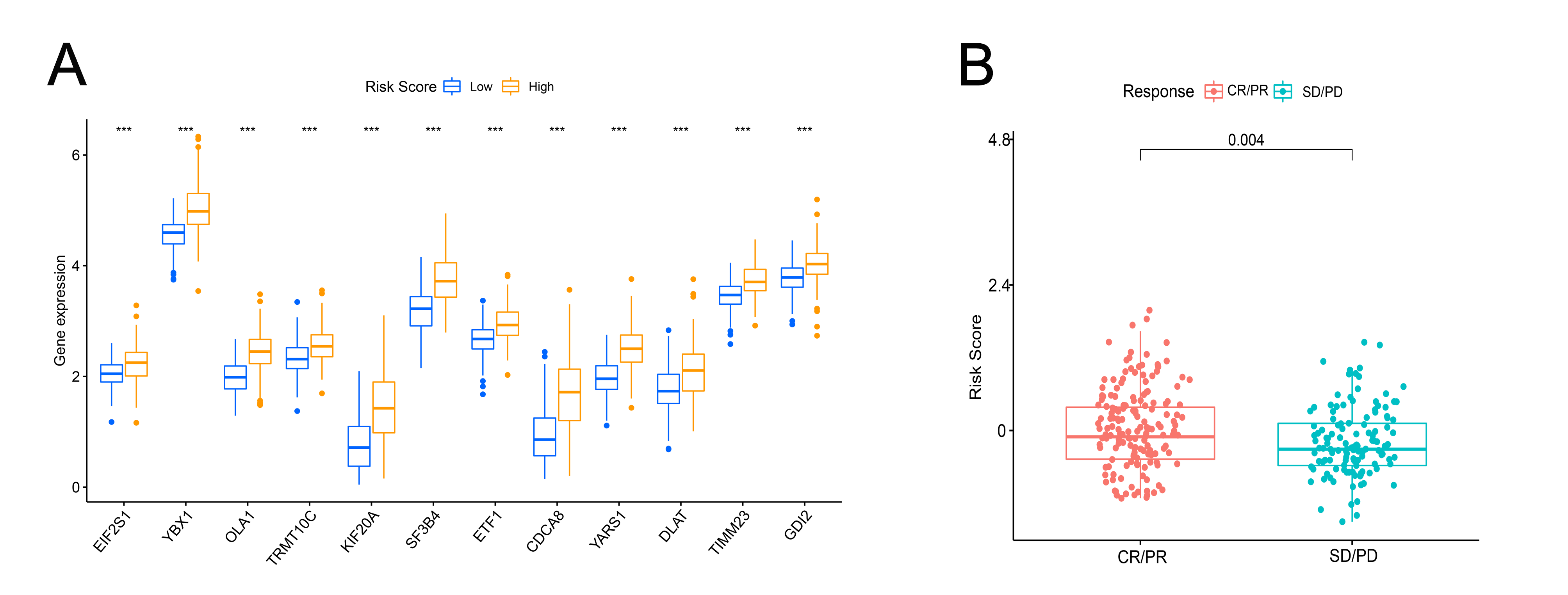

Supplement: Supplementary Figure 3 — (A) The expression levels of 12 hub genes between risk groups. (B) The comparison of risk score for patients with stable disease or progressive disease (SD/PD) and patients with complete response or partial response (CR/PR) in the IMvigor210 cohort. [file Image_3.tif]
